# Evidence of nNOS and ChAT positive phenotypes in nervous ganglia of the retrostyloid space

**Published:** 2012-12-25

**Authors:** F Andrei, AC Didilescu, MC Rusu

**Affiliations:** * Department of Anatomy, Faculty of Medicine, "Victor Babeş" University of Medicine and Pharmacy, Timişoara, Romania; ** Discipline of Embryology, Faculty of Dental Medicine, “Carol Davila" University of Medicine and Pharmacy, Bucharest, Romania; *** Discipline of Anatomy, Faculty of Dental Medicine, “Carol Davila" University of Medicine and Pharmacy, Bucharest, Romania; **** MEDCENTER – Center of Excellence in Laboratory Medicine and Pathology

**Keywords:** cranial nerves, nodose ganglion, petrosal ganglion, immunohistochemistry

## Abstract

The cholinergic and nitrergic phenotypes in human fetal ganglia (inferior) of the glossopharyngeal and vagus nerves were overlooked in basic research. Lack of a positive neuronal NO synthase (nNOS) phenotype in the inferior vagal fetal ganglion was recently suggested to be an individually variable phenotype. Choline acetyltransferase (ChAT) was not evaluated previously in ontogenesis. We aimed to evaluate these phenotypes in human midterm fetuses. Samples from five specimens with gestational ages varying from 4 to 6 months were used. Immunohistochemistry for nNOS, ChAT, neurofilaments, and S100 protein was performed. Neuronal somata were positively stained for nNOS, ChAT and neurofilaments in the inferior glossopharyngeal and vagal ganglia. S100 protein distinctively labelled the satellite glial cells ensheating the respective neurons. In human midterm fetuses vagal and glossopharyngeal inferior ganglia are nitrergic and cholinergic. To evaluate a functional role of these phenotypes in ontogenesis, the specific anatomic circuits should be further checked. Differences in immune labelling should be evaluated by use of similar antibodies from different manufacturers.

## Introduction

Nitric oxide (NO) is produced in many different cells and is involved in the regulation of physiologic events, such as inflammation, vascular tone, and metabolism. Depending on cell type, NO is formed in an enzymatic reaction catalyzed by one of the three isoforms of NO synthase (NOS). Neuronal NOS (nNOS) and endothelial NOS (eNOS) are constitutive, and produce small amounts of NO after stimuli that raise intracellular calcium concentrations. Agents such as cytokines express the third isoform, inducible NOS (iNOS), after induction [**1**]. The nNOS isoform is required for the thermoregulation and participates in the production of fever in rats [**2**]. NO may act on vascular smooth muscle, decreasing vascular tone, stimulating thermogenesis in brown fat, and modulating neuroendocrine function [**2**]. 

Choline acetyltransferase (ChAT), the enzyme responsible for the biosynthesis of acetylcholine, is currently the most specific indicator for the monitoring of the functional state of cholinergic neurones in the peripheral nervous system [**3**]. 

It was shown that neurons in the inferior vagal ganglion (nodose ganglion) are ChAT immunoreactive [**4**]. However, the ChAT phenotype was not previously investigated in human fetal cranial ganglia.

Neuronal NO synthase immune reactivity in human fetal cranial ganglia was searched in a single study, recently performed [**5**]. Unexpectedly, no nNOS immune reactivity was found in the fetal inferior vagal ganglion [**5**]. However, nNOS is a marker for non-cholinergic parasympathetic nerves, and nNOS-positive neurons were demonstrated in the human tracheal wall, which is supplied by vagal fibers [**6**].

Therefore, we aimed to evaluate the nNOS and ChAT immune phenotypes in fetuses samples of retrostyloid space nervous ganglia: inferior vagal and glossopharyngeal.

## Materials and methods

Autopsy samples including the upper pharynx and the parapharyngeal spaces were dissected out in blocks from five human midterm fetuses with ages varying from 4 to 6 gestational months. Samples were drawn immediately post abortion. The Bioethics Committee of “Carol Davila" University of Medicine and Pharmacy, Bucharest, Romania granted approval for the present study. 

The collected samples were fixed for 24 hours in buffered formalin (8%), and then were processed with an automatic histoprocessor (Diapath, Martinengo, BG, Italy) with paraffin embedding. Sections were cut manually at 3 μm, and were mounted on SuperFrost ® electrostatic slides for immunohistochemistry (Thermo Scientific, Menzel-Gläser, Braunschweig, Germany). Histological evaluations used 3 μm thick sections stained with haematoxylin and eosin.

Anti-neurofilaments triplet (clone NE-14, BioGenex, Fremont, CA, USA, RTU), anti-S100 protein cocktail (clone 15E2E2 + 4C4.9, Biocare Medical PME 089 AA, Biocare Medical, Concord, CA, USA, 1:50), anti-ChAT (choline acetyltransferase, clone 38B12, Novocastra-Leica, Leica Biosystems Newcastle Ltd, Newcastle Upon Tyne, UK, 1:50) and anti-nNOS (clone NOS-125, Novocastra-Leica, Leica Biosystems Newcastle Ltd, Newcastle Upon Tyne, UK, 1:50) primary antibodies were used. Sections were deparaffinised, rehydrated and rinsed in phosphate-buffered solution (PBS) at pH 7.4. Retrieval by cooking in specific buffer was completed: 0.01 M citrate retrieval solution (pH 6, 20 minutes). Appropriate endogenous blocking peroxidase was completed before immunolabeling (0.1% BSA in PBS). Sections incubated with non-immune serum served as negative controls. Sections were counterstained with haematoxylin. 

The microscopic slides were analyzed and micrographs were taken and scaled using a Zeiss working station which was described elsewhere [**7**]. 

## Results

**Fig. 1:**
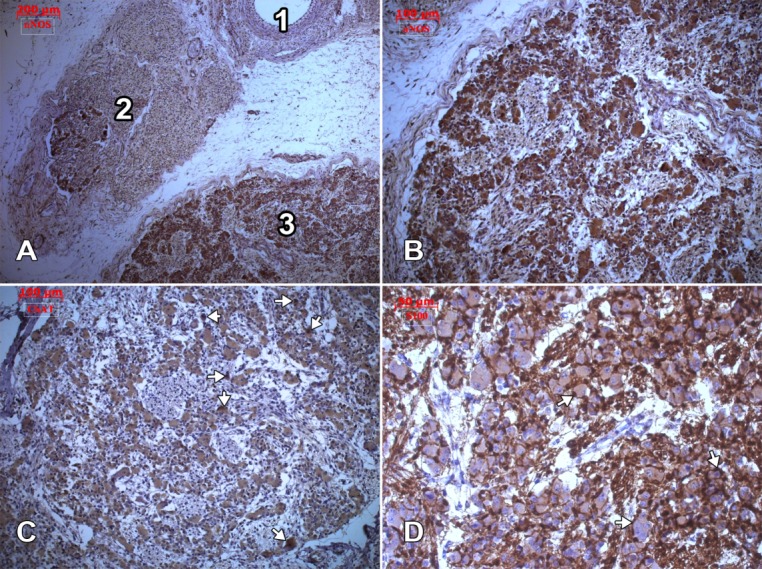
Transverse cuts of the retrostyloid space. Immune labelling for nNOS (A, B), ChAT (C) and S100 protein (D). 1. internal carotid artery; 2. glossopharyngeal nerve, inferior ganglion; 3. inferior vagal ganglion (also depicted in B, C and D).

 On transverse cuts, the neurovascular content of the retrostyloid space was identified anteriorly to the transverse process of the first cervical vertebra: the internal carotid artery, internal jugular vein, the glossopharyngeal, and vagus nerves (Fig. 1A). 

The inferior vagal ganglion was encapsulated in a fibrous capsule, and it was placed posteriorly to the internal carotid artery. The glossopharyngeal nerve was postero-medial to the internal carotid artery; it presented on its course the lower pole of its inferior ganglion, and it consisted of distinctive nerve bundles.

Both ganglia, the inferior vagal ganglion (nodose ganglion) and the inferior ganglion of the glossopharyngeal nerve (petrosal ganglion), presented immune positive reactions of the neuronal somata when labelled with antibodies against the neurofilaments triplet, nNOS and ChAT (**Fig. 1A-C**), in all samples. Usually, each neuron was completely surrounded by a capsule built-up by satellite glial cells which were S100 protein immune positive (**Fig. 1D**).

## Discussion

 First time evidence of a positive immune phenotype for nNOS and ChAT of neurons in the inferior vagal and glossopharyngeal ganglia, is hereby provided in midterm fetuses. Concerning the nNOS phenotype, the only similar study that can be referred is that of Kiyokawa et al. (2012) in which the authors found the inferior vagal ganglion as being nNOS phenotype [**5**], by use of antibodies from a different manufacturer. As the authors state, "strangely, we found no nNOS reactivity in the IVG" [**5**]. The unexpected nNOS negative phenotype was related there to the individual variation in immunohistochemical expression [**5**]. However, the sources of antibodies should be also considered, and larger lots should be evaluated for the NOS phenotype by simultaneous use of antibodies from different manufacturers.

It resulted here that the inferior glossopharyngeal and vagal ganglia are cholinergic, as well as nitrergic. This is a similar phenotype to that identified in cardiac ganglia [**8**].

It seemed natural to find a nNOS phenotype in ganglia of cranial nerves known to be related to autonomic functions. Neurons with immune reactivity to NOS were found to be distributed in various mucosae [**9-12**]. Afferent neurons in the nodose ganglia that distribute to the stomach were demonstrated to express nNOS; anterograde-traced vagal endings are, however, nNOS negative, indicating that NOS is not transported peripherally [**13**]. A subpopulation of enteroendocrine cells in the mucosa were nNOS positive, which were found anatomically in close apposition with mucosal vagal afferent endings. This indicates an inhibitory neuromodulatory role of epithelial NO, which targets a distinctive population of vagal afferences [**13**]. It was previously shown that microganglia in the carotid body and glossopharyngeal and carotid sinus nerves exhibit NOS- and ChAT-positive immune reactivity, and functionally related NOS-containing neurons are located in the petrosal ganglion [**14**]. 

By immunohistochemistry, neurons within the nodose ganglion were found ChAT-positive, suggestive for choline uptake and an endogenous content of acetylcholine [**15**]. As resulted from the present study, this could be an ontogenetic feature. The presence of ChAT in the sensory nodose projection to the solitary nucleus in medulla oblongata cannot be ruled out [**16**].

It appears so that the nitrergic and cholinergic neuronal phenotypes in the petrosal and nodose ganglia in human midterm fetuses are positive and able to support different functional mechanisms. However, the corresponding anatomic circuits should be completely evaluated for the specific immune phenotypes involved in neural transmission and function. 

**Acknowledgements**

This study was supported by the Sectoral Operational Programme Human Resources Development (SOP HRD), financed from the European Social Fund and by the Romanian Government under the contract number POSDRU/89/1.5/S/64153 (author #3). 

**Conflicts of interest**

The authors confirm that there are no conflicts of interest.
